# Annotations

**Published:** 1891-01-03

**Authors:** 


### Annotations.

**The Living Workmen and The Dead Archbishop.**

Eight representative working men of Sheffield, by special arrangement with the family, carried the Archbishop of York to his grave on Tuesday last. All classes of people who love England will do well to consider this fact. Archbishop Thomson was a great man: great in intellect, in learning, and in moral worth. He filled the place of a Prince in the Church, and he had a princely heart. He admitted the claim of every man, woman, and child in his diocese to his friendship and his paternal kindness. Pride, which is too often the curse of priest and prince alike, took in him the form of a magnanimous humility. He understood the English people among whom he exercised spiritual authority; and he understood the essence of the Christian faith which gave him his authority. Therefore, though he was far removed from the working people in the broad and deep culture of his mind, and in the elevation of his ecclesiastical position, when he went among them, as he constantly did, he surprised them by his kindly simplicity, and won the devotion of their hearts by his untainted sincerity. When such men as Dr. Thomson are in high place misunderstandings cease, and class hatreds die away. The representative workmen of Sheffield did not go at the bidding of their fellows to bury an Archbishop, but to bury a man. Rough people are those same Sheffield working men, but they have hearts, and brains too. The men in high place in Yorkshire and every other English county owe it to themselves, as well as to the memory of Dr. Thomson, to think of the kind of man he was, of the life he led, and of the wholesome fruit it bore among all classes of the people. Long may Christianity, long may science, and long may the State furnish us with men like the late Archbishop for leaders and exemplars.

**Boy and Girl Marriages.**

Will practical statesmen, both in and out of Parliament, pay a little heed to this? A youth of eighteen, named Alfred Gomm, was charged at the West London Police Court, on Monday, with attempting to commit suicide. The boy's mother explained that he was a married man, and that he had no home comforts. His wife had reached the capable and experienced age of sixteen. The magistrate asked the mother what she could expect from a marriage of that kind? The young married man himself was remanded, and handed over to the chaplain for good advice. It never appears to occur to us English people that we ought to tackle our public business in a different way from what we have been accustomed to. A man who travels a strange country in the dark may be excused if he does not advance at the rate of more than a mile an hour. But when the sun is up, and he can see the track clearly, he is expected to make three or four miles an hour at the very least. Several suns have shone upon this country since the days of the ancient Britons and their Druidical priests. Not only has a reasonable religion given its light, but literature has shed its rays, and, since the time of Bacon, science has been carrying all sorts of light into every nook and corner of civilization. Now, if this boy and girl be brought into the daylight, either of religion, or of literature, or of science, they are seen to be fools, and not only fools, but irresponsible fools. But there is no person, and no thing in the world that somebody is not responsible for. Who is responsible for this boy and girl? Their parents? Yes, if their parents had power over them. But they have not, and nobody can properly be charged with responsibility who is not endowed with power. The State, therefore, is responsible for the restraining of the passion-madness of boys and girls like these we are discussing. The State in this country consists of the voters and their representatives. Every one of us therefore must admit a share of responsibility; and that share is to be measured by our intellectual capacity. We appeal to every man of brains to think what is to be done with the boys and girls of the working classes who rush into matrimony as they rush into a theatre, without thought and without care; and we appeal to every man of no brains to hold his tongue and to refrain from arguing on the subject. This is a terrible question. The writer sees a large number of out-patients every week; and it is one of his commonest experiences to find women of twenty or twenty-one who have already two or three children, and women of twenty-six or twenty-seven who have already five or six. The women are thin, bloodless, incapable, and miserable. Their children are small, stunted, rickety, and starved. The homes of these creatures are airless, dirty, and poisonous. What is the general result? That the bulk of the working classes in the large towns are becoming altogether incapable of either physical or mental advancement, and only capable of deeper depths of feebleness, incapacity, and degradation. This is not rhetoric. Some man or men of knowledge and understanding must take up the case of the physical and mental degradation of the masses of people in large towns. The root of the tree must be struck at in early marriages. No workman should be permitted to marry before he is twenty-five, except by special State licence; and no woman of the same class until she is twenty-three. This is no party question! Is there not a man, on one or the other side of politics, who will separate himself from his party and devote himself to the doing of so great a service for the State? Whoever should grapple successfully with this question would take rank among the greatest and noblest statesmen of our own or any other country or time.

**The Vice of Pity.**

Mrs. Lynn Linton has been lecturing the world on "The Vice of Pity." The Rev. S. A. Barnett, rector of St. Jude's, Whitechapel, takes up the cause of the pitiful and the pitied, and defends it *à outrance*. Physiology and sociology present many parallelisms. When any particular human body is out of health in any one of its parts, it is found that the whole body is thereby rendered more or less miserable and inefficient. The ill person feels it desirable to have his illness radically treated and cured. He knows two sorts of doctors: there is the man who is rough and thorough, and the man who is kind and thorough. He prefers the man who is kind and thorough, and that for two reasons. He finds, first of all, that he himself can work better with a kind man than with a rough one; and, secondly, that the disease under which he is labouring is not only cured more pleasantly, but also more quickly by kind thoroughness than by rough thoroughness. The body sociological, or, in other words, the community, is as decidedly a unit as the individual man. If any part of it suffers, that part cannot suffer alone; the whole body shares in the suffering, in the misery, and in the inefficiency. This is as plain to the man who has eyes as the dome of St. Paul's is to the balloonist who is sailing over London. The man or women who cannot see it ought not to have the boldness to say another word on the subject. The kind of treatment that is found best for the individual has been proved to be also best for the mass. Many states have been rough and thorough in their dealings with poverty and distress. Such states with all their thoroughness have only succeeded in thoroughly failing. This country has had the originality for a couple of centuries to be kind and thorough in its dealings with the poor, the sick, and the distressed. The result has amply justified the method of kindness and thoroughness. Mr. S. A. Barnett admits that certain so-called "pitiful" persons do some harm. But as one of the most experienced and capable philanthropists in London he holds that the harm done by indiscriminate pity is as a single drop in the shower compared with the good done by discriminating pity. The problem is very simple: the inefficient and the poor are here with us; they always have been here; they always will be here; they are as an eating ulcer on the surface of the social organism. The ulcer cannot be cut out; for if it were, another would appear elsewhere. It can only be healed up to a certain point, and the best principles of treatment are found to be those that soothe pain and encourage healthy action. Leave the ulcer alone and it spreads and destroys the body; treat it with irritants and escharotics, and it spreads all the more. We make no appeal to pity; our appeal is to experience, to science, and to sound physiological principles. There is no such thing as vice in true pity; it is a healing balm. The despisers, the repressors, the hard and stern economists, as they call themselves, are simply the unskilful doctors whose methods have proved complete failures, and who try to hide their own want of success by attacking the better methods of their neighbours. Let a single example suffice: Mrs. Lynn Lynton holds up "pity" to scorn as a "vice." Mr. S. A. Barnett defends both the pity and the pitiful. Whether of the two has proved in practice the more successful doctor in dealing with the ills of the social organism?

